# Seroprevalence of *Mycobacterium* *avium* subsp. *paratuberculosis* in Dairy Cattle in Khartoum State, Sudan

**DOI:** 10.3390/vetsci7040209

**Published:** 2020-12-21

**Authors:** Wisal A. Elmagzoub, Nabawia M. Adam, Sanaa M. Idris, Mohamed E. Mukhtar, Sanaa A. Abdelaziz, Julius B. Okuni, Lonzy Ojok, Ahmed Abd El Wahed, ElSagad Eltayeb, Ahmed A. Gameel, Kamal H. Eltom

**Affiliations:** 1 Unit of Animal Health and Safety of Animal Products, Institute for Studies and Promotion of Animal Exports, University of Khartoum, Shambat 13314, Khartoum North, Sudan; wisalelmagzoub@gmail.com (W.A.E.); sanaaidris15@gmail.com (S.M.I.); 2 Department of Biology and Biotechnology, College of Applied and Industrial Sciences, University of Bahri, Alkadaro 13316, Khartoum North, Sudan; 3 Department of Microbiology, Faculty of Veterinary Medicine, University of Khartoum, Shambat 13314, Khartoum North, Sudan; nabawiavet02011@gmail.com (N.M.A.); sabdelaziz262@gmail.com (S.A.A.); 4 Department of Pathology, Faculty of Veterinary Medicine, University of Khartoum, Shambat 13314, Khartoum North, Sudan; aargameel@hotmail.com; 5 Department of Agricultural Extension and Rural Development, Faculty of Agriculture, University of Khartoum, Shambat 13314, Khartoum North, Sudan; elgadidwas@yahoo.com; 6 College of Veterinary Medicine, Animal Resources and Biosecurity (COVAB), Makerere University, P.O. Box 7062, Kampala, Uganda; jbokuni@gmail.com (J.B.O.); lonzyo@yahoo.com (L.O.); 7 Department of Pathology, Faculty of Medicine, Gulu University, P.O. Box 166, Gulu, Uganda; 8 Institute of Animal Hygiene and Veterinary Public Health, Faculty of Veterinary Medicine, University of Leipzig, an den Tierkliniken 43, D-04103 Leipzig, Germany; 9 Faculty of Medicine, Al Neelain University/Ibn Sina Specialised Hospital, Alamarat, Street 17–21, 12217 Khartoum, Sudan; sagadgady@yahoo.com

**Keywords:** *Mycobacterium avium paratuberculosis*, seroprevalence, cattle

## Abstract

Paratuberculosis, caused by *Mycobacterium avium* subspecies *paratuberculosis* (MAP), is a chronic wasting disease mainly of domestic and wild ruminants. It occurs worldwide, causing significant economic losses through decreased productivity, low fertility, increased cull rates and mortality. It is listed by the OIE (World Organization for Animal Health) as a disease of concern to trade in animals. Prevalence of this disease can be studied by detecting anti-MAP antibodies by Enzyme linked immunosorbent Assay (ELISA). The aim of this study was to investigate the current prevalence of MAP infection in cattle in Khartoum State. The overall apparent prevalence of MAP infection was found to be 6.3% and 18.9% at animal and herd levels, respectively. All seropositive animals were cross-bred females of good body condition; most of them (>90%) were >3 years old and >50% were from medium-sized herds in Omdurman. No significant association (*p* > 0.05) was found between seropositivity and animal herd size. The prevalence of MAP infection in Khartoum State is still low to medium compared to other parts of the world, but it is comparable to those reported from other African countries. Further studies with the view of designing nationwide surveys in domestic ruminants and camels in other states of the country are needed for establishing control programmes.

## 1. Introduction

Paratuberculosis (PTB), also known as Johne’s disease, is a chronic enteritis caused by *Mycobacterium avium* subsp. *paratuberculosis* (MAP) affecting ruminants and wild mammals worldwide [1,2]. It is listed by the OIE (World Organization for Animal Health) as a disease of concern to trade in animals. The disease affects animals of all ages, manifesting as different clinical stages [3,4,5], causing decreased productivity, low fertility and increased susceptibility to infections [6]. Moreover, MAP is suspected to be involved in the aetiology of Crohn’s disease, a chronic diarrhoeal inflammatory bowel disease of humans, which shares some features with PTB [3,7]. Due to the serious economic impact of PTB on livestock production, control of MAP is very important wherever it occurs. This requires early detection of infected livestock and sufficient information of the rate of infection and associated risk factors in the population.

Diagnosis of PTB is based on one or more of the following: isolation of MAP or detection of its DNA in tissues or faeces, detection of MAP antibodies and culture to isolate the organism. The suitability and sensitivity of the tests depend on the clinical stage of the disease [8,9]. Silent infection can be detected only by histopathology or MAP tissue culture, which takes a long time [10,11]. Enzyme-Linked Immuno-Sorbent Assay (ELISA) is a favourable and commonly used serological test to detect MAP antibodies in serum and milk in the subclinical stage of MAP infection and afterwards [3,10]. ELISA’s sensitivity may reach a maximum of 50% [8] and its specificity can be improved through pre-treatment of samples with *Mycobacterium phlei* to absorb non-specific antibodies [12] and minimize possible false positives.

Knowledge of the global distribution of PTB is important for establishing control programmes. The prevalence of PTB has been reported from different countries, mainly for bovine PTB. Herd-level prevalence of up to 75.8% was reported in the Caribbean and Latin America [13], of >50% in Europe and North America [14] and of 20.35–41.7% in Asia [15,16]. Animal-level prevalence ranging from 2.31% to 29.8% was also reported in Asia [15,16,17,18]. In Africa, reports on MAP infections in animals are rather scant. Available published data show herd prevalence of 45.2% and 13% in Egypt and Uganda, respectively [19,20], and animal-level prevalence of 9% in Uganda [20] and 5.26% in Tanzania [21]. Although PTB was first diagnosed in cattle and goats in Sudan more than 50 years ago [22,23], there has been little research and few reports about its incidence and prevalence. The first report on bovine PTB in the Sudan was from Khartoum, with 53% prevalence, based mainly on the diagnosis of the clinical disease [23]. A more recent study on bovine PTB in Khartoum State by Mohammed et al. in 2010 revealed 66.7% and 10.2% prevalence at the herd and animal levels, respectively [24], but it was limited since it involved only a few farms. The present study aimed at evaluating the current situation of PTB in dairy farms in Khartoum State by increasing the number of herds in a wider area.

## 2. Materials and Methods

### 2.1. Ethical Statement 

Ethics approval for the study was waived by the Research Board of the Faculty of Veterinary Medicine and the Scientific Committee of the Institute for Studies and Promotion of Animal Exports, University of Khartoum. The samples were taken as part of routine diagnosis by authorized veterinarians according to the national veterinary and animal welfare regulations.

### 2.2. Study Population

**Figure 1:**
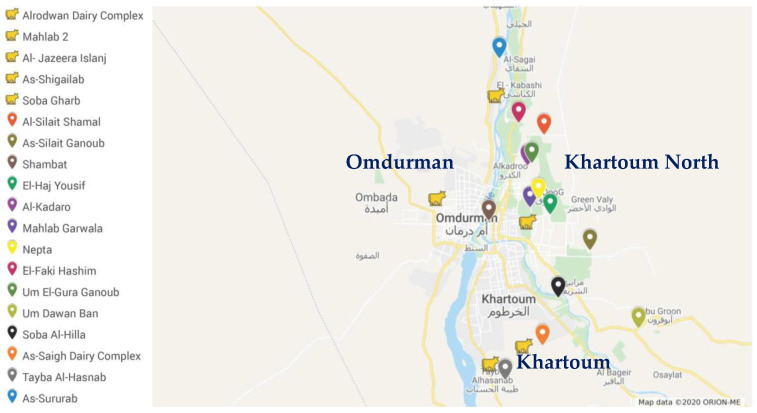
Locations (N = 19) of 37 dairy farms sampled for the seroprevalence of *Mycobacterium avium* subsp. *paratuberculosis* (MAP) in Khartoum State. Locations with the cow symbol are those with MAP +ve herds. The map was created from Google Maps® using Google’s account of Kamal H. Eltom.

Animals included in this study were local (Butana/Kenana) and cross-bred (local × Friesian) cattle of both sexes and different ages selected through stratified random sampling from different dairy herds in three major areas in Khartoum State (Figure 1): 11 herds in Khartoum, 17 in Khartoum North and 9 in Omdurman, based on cooperation from animal owners/herders. Most of the animals were of good body condition.

### 2.3. Enzyme Linked Immuno Sorbent Assay (ELISA) Procedure

The obtained sera were tested for anti-MAP antibodies using the IDEXX Paratuberculosis Screening Ab Test (IDEXX laboratories Inc. Westbrook, USA) according to the manufacturer’s instructions. In brief, the test samples and the provided controls were diluted 1:20 with the provided buffer containing *Mycobacterium phlei* and left (as a pre-incubation) for 35 min. The plate coated with MAP antigen was incubated for 45 min with 100 µL of the diluted test samples and controls. The plate was then washed thrice, 100 µL of conjugate was added, plates were left for 30 min and washed as before; 100 µL of TMB solution was added and left for 10 min, followed by 100 µL of stop solution. The optical density of the plates was read at 450 nm in a plate reader. According to manufacturer’s guidelines, the results were validated and then interpreted as positive or negative.

### 2.4. Statistical Analysis

The results were analysed using SAS software version 9 (SAS Institute Inc., Cary, NC, USA). The data obtained were compared using the Pearson chi-squared test. 

## 3. Results and Discussion

In this study, antibodies against MAP were demonstrated by ELISA in 7 out of 37 dairy herds tested in Khartoum State with an overall herd prevalence of 18.9% and animal-level prevalence of 6.3%. Three of the positive herds were in Omdurman, which also had the highest prevalence at the animal level (33.3%); two positive herds were found in each of the other two localities. A summary of the results is presented in Table 1. As the maximum sensitivity of ELISA is about 50%, the true estimation of seroprevalence could be higher than the obtained results. However, ELISA-positive animals may or may not be MAP shedders; antibodies could be produced before shedding started or years after [5], but precautions should be taken to minimize the transmission of infection within the herd.

All seropositive animals were cross-bred females of good body condition; most (>90%) of them were above three years old and >50% were from a medium-size herd (50–100 heads). The chronic nature of MAP infection and the long incubation period render the infected animals apparently healthy for years before showing overt symptoms of the disease [25], which may explain our findings. The numbers of local cattle in the farms investigated was small, and none of the tested animals was seropositive. Local breeds are poor milkers compared to exotic breeds or their crosses, and, therefore, limited numbers of selected cows were included. However, PTB surveys should also include pastoral local herds in Sudan. It is interesting that the chi-squared test showed no significant association between these descriptive animal data and serostatus (Table 2).

The only reliable report from Sudan on the seroprevalence of bovine PTB available to compare the current results with is that of Mohammed et al. [24], who reported 66.7% and 10.2% prevalence at the herd and individual animal levels, respectively, in Khartoum State. These rates are higher than those reported here, but our study is more representative due to involving greater number of herds distributed over a wider area of Khartoum State (Figure 1). Moreover, they involved clinical cases; when these are excluded, the apparently healthy animals would reveal a comparable animal-level prevalence (6.9%). Contrary to the current results, they reported the lowest seropositivity in Omdurman. Possible explanations for this increase in seropositivity of herds in Omdurman could be the establishment of infection through time because of close proximity of dairy farms and stability of locations of many dairy compounds for the last three decades, while in Khartoum and Khartoum North, the dairy compounds’ locations had been changed due to the encroachment of urban areas.

It is obvious that PTB in Sudan is poorly documented. Similarly, reports from other African countries on MAP infections in animals are rather scant [26]. However, the prevalence rates, at herd or individual animal levels, reported from Egypt [19], Uganda [20] and Tanzania [21] are reasonably comparable with the present results. Global prevalence of PTB is high, and the disease is well documented in many developed countries, where it causes tremendous economic losses. The prevalence rates of PTB obtained in this study seem to be low compared to those mentioned globally [13,15,16,17,18].

Generally, variation between the results obtained by various authors may be influenced by the stage of infection, animal age, shedding level of the organism, lactation, antibody concentration and varying sensitivity among different ELISAs [5,8,25,27]. These should be considered when comparing results on PTB prevalence.

It has been stated that ELISA is more precise in determining herd-level prevalence [8]; however, the combination of ELISA with culture or molecular assays for the screening of MAP infection would be more reliable in investigating both animal- and herd-level prevalence.

## 4. Conclusions

The present study confirms that MAP infection occurs in dairy cattle in Khartoum State at a similar prevalence to other African countries. However, studies that are more detailed are needed to establish a better view on the prevalence, which will allow the establishment of control programmes. This will allow a more optimal and sustainable production system for Sudan and Africa, enabling better food supply for the people.

## Figures and Tables

**Table 1:** Prevalence of MAP antibodies in dairy cattle in Khartoum State.

Area	Animal Level			Herd Level		
	No. Tested	Positive		No. Tested	Positive	
		No.	%		No.	%
Khartoum	51 *	2	3.9	11	2	18.2
Khartoum North	78	4	5.1	15	2	13.3
Omdurman	45	5	11.1	9	3	22.2
Total	175	11	6.3	37	7	18.9

* Includes seven animals of local breed; all were negative.

**Table 2:** Chi-squared test results for associations between cattle age, body condition, herd size, sex and prevalence of MAP antibodies in Khartoum State.

Result		Age			Body Condition			Herd Size				Sex		
		>3	<3	Total	Emaciated	Good	Total	L	M	S	Total	Female	Male	Total
Negative	Count	119	45	164	28	136	164	44	81	39	164	137	27	164
	% of total	68.0	25.7	93.7	16	77.7	93.7	24.9	46.3	22.3	93.7	78.3	15.4	93.7
Positive	Count	10	1	11	0	11	11	2	6	3	11	10	1	11
	% of total	5.7	0.6	6.3	0.0	6.3	6.3	1.2	3.4	1.7	6.3	5.7	0.6	6.3
Total	Count	129	46	175	28	147	175	46	87	42	175	147	28	175
	% of total	73.7	26.3	100.0	16.0	84.0	100.0	26.3	49.7	24.0	100.0	84.0	16.0	100.0

The chi-squared test results showed that the association between cattle age and the prevalence of MAP antibodies was not significant (chi-squared value = 1.9, *p* > 0.05); similarly, the association between the cattle body condition and the prevalence of such antibodies was insignificant (chi-squared value = 2.2, *p* > 0.05). Neither sex nor the herd size was significantly associated with the prevalence of MAP antibodies (chi-squared value = 0.5, *p* > 0.05) and (chi-squared value = 0.38, *p* > 0.05), respectively. L (Large herd), M (Medium herd) and S (Small herd).
